# Predicting Cancer-Specific Survival Among Patients With Prostate Cancer After Radical Prostatectomy Based on the Competing Risk Model: Population-Based Study

**DOI:** 10.3389/fsurg.2021.770169

**Published:** 2021-11-26

**Authors:** Xianghong Zhou, Shi Qiu, Kun Jin, Qiming Yuan, Di Jin, Zilong Zhang, Xiaonan Zheng, Jiakun Li, Qiang Wei, Lu Yang

**Affiliations:** Department of Urology, National Clinical Research Center for Geriatrics and Center of Biomedical Big Data, Institute of Urology, West China Hospital of Sichuan University, Chengdu, China

**Keywords:** competing risk analyses, prostate cancer, radical prostatectomy, post-surgery, predicting model

## Abstract

**Introduction:** We aimed to develop an easy-to-use individual survival prognostication tool based on competing risk analyses to predict the risk of 5-year cancer-specific death after radical prostatectomy for patients with prostate cancer (PCa).

**Methods:** We obtained the data from the Surveillance, Epidemiology, and End Results (SEER) database (2004–2016). The main variables obtained included age at diagnosis, marital status, race, pathological extension, regional lymphonode status, prostate specific antigen level, pathological Gleason Score. In order to reveal the independent prognostic factors. The cumulative incidence function was used as the univariable competing risk analyses and The Fine and Gray's proportional subdistribution hazard approach was used as the multivariable competing risk analyses. With these factors, a nomogram and risk stratification based on the nomogram was established. Concordance index (C-index) and calibration curves were used for validation.

**Results:** A total of 95,812 patients were included and divided into training cohort (*n* = 67,072) and validation cohort (*n* = 28,740). Seven independent prognostic factors including age, race, marital status, pathological extension, regional lymphonode status, PSA level, and pathological GS were used to construct the nomogram. In the training cohort, the C-index was 0.828 (%95CI, 0.812–0.844), and the C-index was 0.838 (%95CI, 0.813–0.863) in the validation cohort. The results of the cumulative incidence function showed that the discrimination of risk stratification based on nomogram is better than that of the risk stratification system based on D'Amico risk stratification.

**Conclusions:** We successfully developed the first competing risk nomogram to predict the risk of cancer-specific death after surgery for patients with PCa. It has the potential to help clinicians improve post-operative management of patients.

## Introduction

Prostate cancer (PCa) is one of the most common genitourinary tumors. In 2020, it is estimated to cause 33,330 deaths in the United States (1). Radical prostatectomy (RP) has been confirmed as an effective primary treatment for patients with localized PCa (2). However, although with the advancement of surgical techniques, a large number of patients undergoing RP have obtained survival benefits, there are still about 25% of patients who will develop biochemical recurrence, distant metastasis, or even death caused by PCa (3–6). At present, it is still controversial as to which kind of patients need to receive active post-operative adjuvant treatment or receive conservative watchful waiting (7). It is important to identify the patients with a higher risk of recurrence or death, and they may benefit more from post-operative adjuvant treatments.

Some research teams have developed tools to stratify the risk of recurrence or death for PCa patients. For example, D'Amico risk stratification, CAPRA Scoring System, and Stephenson nomogram are commonly used in clinical (8–10). These tools mainly used several clinicopathological parameters such as prostate specific antigen (PSA) level, clinical stage, Gleason Score (GS), and pathologic extent to predict the prognosis. However, these tools are still flawed. They are mainly developed based on a small number of patients, the weight between the various prognostic factors is not clear enough and some studies have pointed out that their prediction accuracy is often <70% (11, 12). In addition, many patients with PCa are elderly people with many comorbidities, and they are more likely to die from cardiovascular disease, infection, or other non-tumor factors. Therefore, it is more difficult for researchers to accurately determine the prognosis of patients (13, 14).

To circumvent these defects, with the approach of competing risk analyses, we evaluated the factors affecting prostate cancer-specific survival (CSS) at a large cohort. Furtherly we developed a prognosis nomogram and construct a risk stratification that may have potential clinical implications to help clinicians identify the patients with a high risk of cancer-specific death after RP.

We present the following article in accordance with the TRIPOD Checklist.

## Methods

### Patient Selection

**Figure 1:**
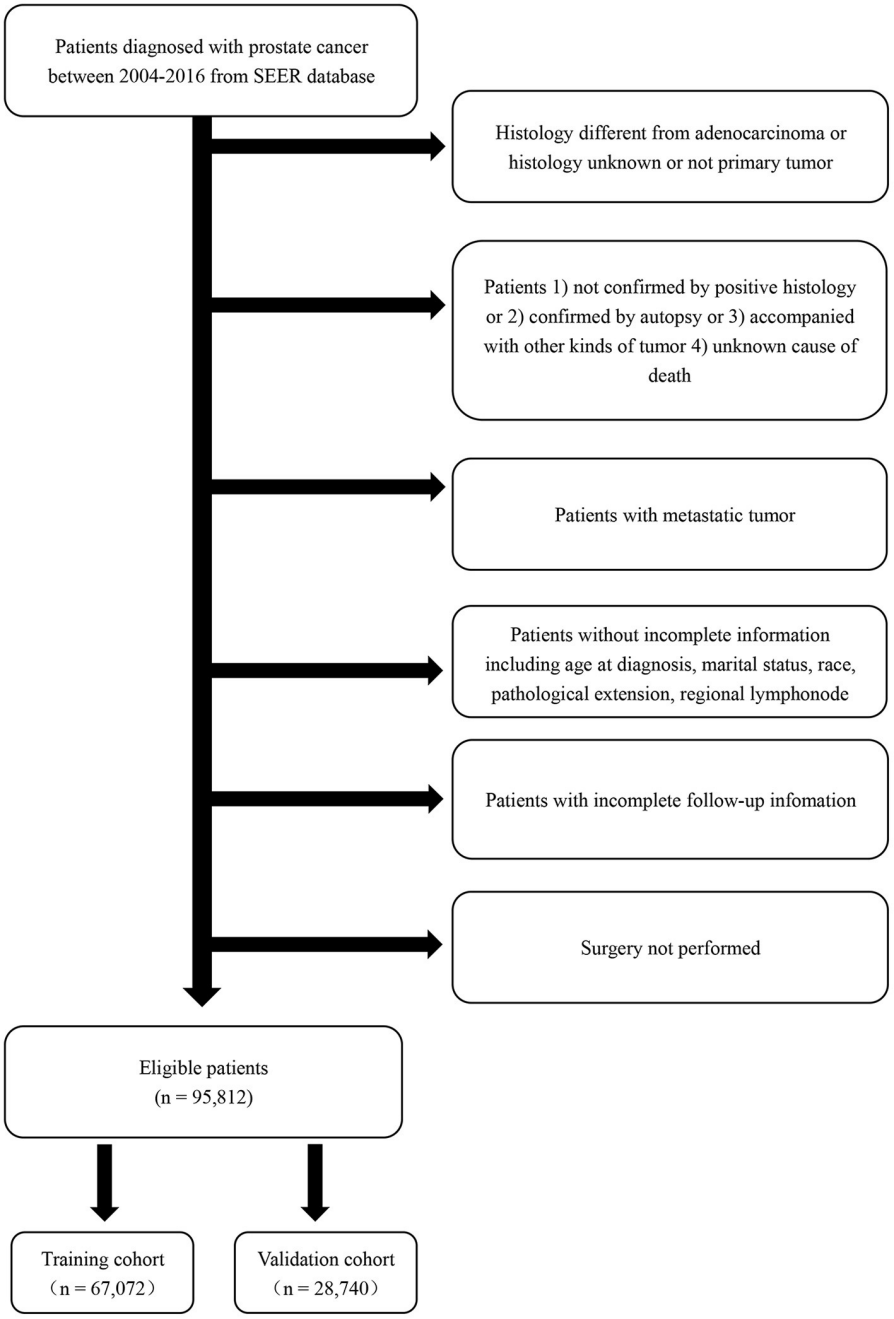
Flowchart describing the inclusion and exclusion criteria of patients in the Surveillance, Epidemiology, and End Results (SEER) database, 2004–2016.

All patients' information was obtained from The Surveillance, Epidemiology, and End Results (SEER) database (2004–2016). SEER database is a public cancer dataset made up of 18 population-based cancer registries. It has covered about 25% population of the United States (14). From the SEER database, patients with a diagnosis of adenocarcinoma of the prostate (International Classification of diseases-O-3 code: C61.9) between 2004 and 2016 were selected. Inclusion and exclusion criteria were shown in the flowchart in detail (Figure 1). With a ratio of 7:3, all the patients were randomly divided into the training cohort and validation cohort.

### Variables and Endpoint

**Figure 2:**
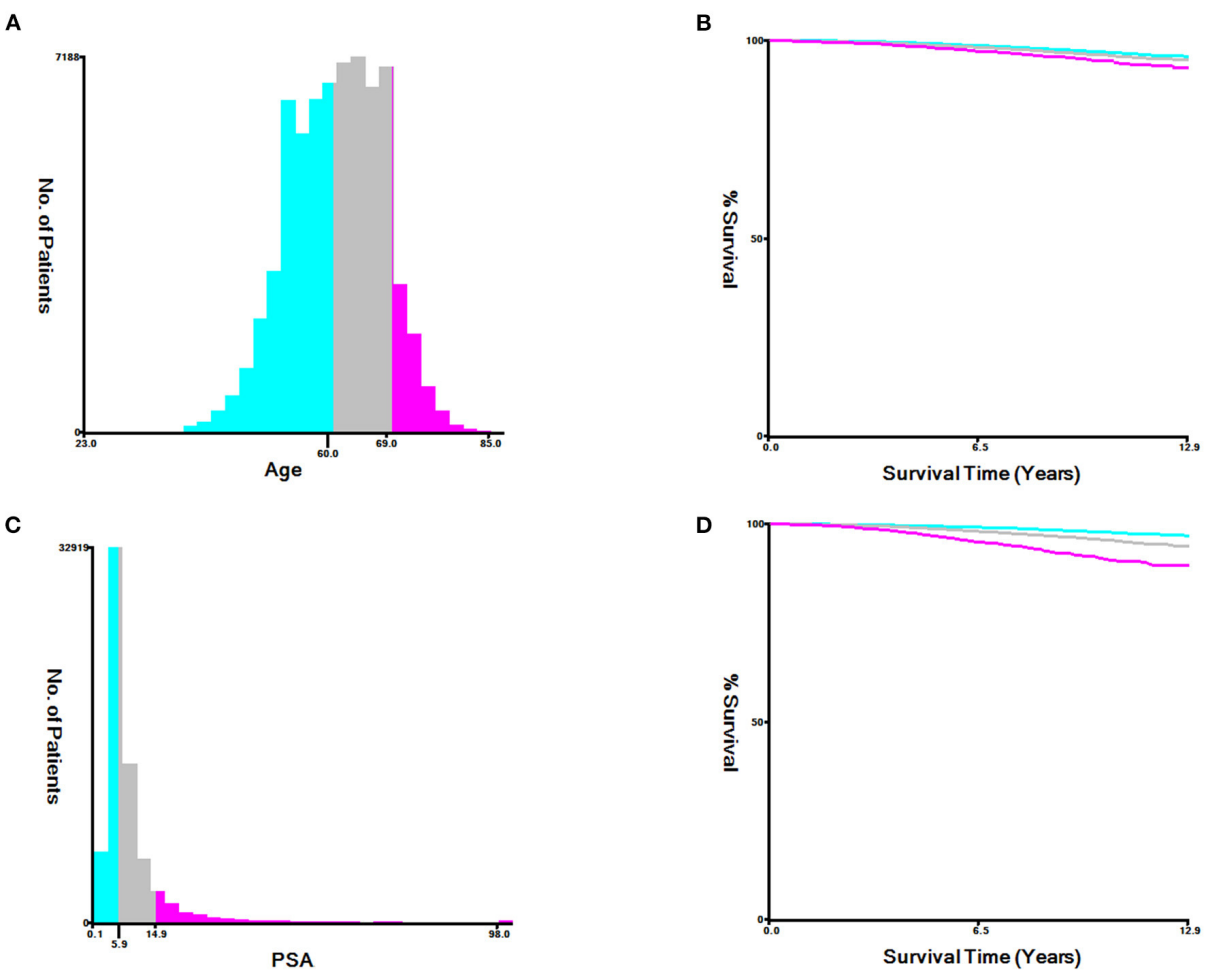
Determination of the optimal cut-off values of age at diagnose **(A,B)**, and prostate specific antigen (PSA) level **(C,D)**. The Optimal cut-off values were identified by the X-tile software according the difference of cancer-specific survival outcomes.

For each patient, the information extracted from the SEER database included age at diagnosis, marital status, race, pathological extension, regional lymphonode status, PSA level, pathological GS, and follow-up information. For continuous variables including age at diagnosis and PSA level, X-tile software (Yale University, USA) was used to assess the optimal cut-off values by the minimal *p*-value approach (15) (Figure 2). The optimal cut-off values for age at diagnosis were ≤ 5.9, 6.0–14.9, >14.9. The optimal cut-off values for PSA level were ≤ 5.9, 6.0–14.9, >14.9.

Cancer-specific death (CSD) was used as the primary endpoint. CSD was measured by all deaths caused by prostate cancer, complications of treatments, or unknown processes in patients with active tumors. Other cause-specific death (OCSD) was measured by all deaths caused by non-cancer events and seen as the competing event of the CSD. Follow-up time was defined as the time between the first treatment and the patient's death or last follow-up.

### Statistical Analysis

For categorical variables, a χ^2^-test was used to evaluate the difference between the training cohort and validation cohort, and the results were presented as the frequency with its proportion. In the training cohort, we estimated the cumulative incidence function (CIF) for CSD and tested the survival differences by Gray's test to discover potential prognostic variables with a *p*-value < 0.05. Subsequently, we performed competing risk multivariable analyses based on the Fine and Gray's proportional subdistribution hazard approach to identify these independent prognostic variables with a *p*-value < 0.05. All the independent prognostic factors were selected to construct a nomogram to predict 5-year CSD probabilities for PCa patients after RP.

To validate the performance of the nomogram, in both two cohorts, the discrimination of the nomogram was assessed by Harrell's concordance index (C-index). The value of C-index ranged from 0.5 to 1, and a higher C-index means better discrimination for the prediction model (16). Besides, the calibration curve with 1,000 resamples of bootstrapping was used to compare the predicted survival outcome with the actual survival outcome. The closer the calibration curve was to the standard curve, the closer the survival outcome predicted by the nomogram was to the actual survival outcome (17).

In addition, we developed a risk stratification based on the nomogram risk score. The cut-off values of risk scores were determined using X-tile software. Then we compare the discrimination abilities of the risk stratification with the European Association of Urology (EAU) risk stratification based on D'Amico stratification (2).

The statistical software R (version 3.4.3, The R Foundation) was used in the above statistical analyses. A *p*-value < 0.05 was considered statistically significant.

## Results

### Patient Characteristics

Finally, a total of 95,812 eligible patients were included in this study. Among them, 67,072 patients were assigned to the training cohort, while 28,740 patients were assigned to the validation cohort. Table 1 showed the characteristics of patients in detail. Between the training cohort and validation cohorts, there were no statistically significant differences except for the age at diagnosis.

**Table 1:** Descriptive characteristics of 95,812 prostate cancer patients undergoing radical prostatectomy between 2004 and 2016 from the Surveillance Epidemiology and End Results database.

**Variable**	**Primary cohort** **(***n*** = 95,812)**		**Training cohort** **(***n*** = 67,072)**		**Validation cohort** **(***n*** = 28,740)**		
	**Number**	**%**	**Number**	**%**	**Number**	**%**	***p*-value**
**Age**							<0.001
≤ 60	39,159	40.9	26,320	39.2	12,839	44.7	
61–69	45,207	47.2	32,783	48.9	12,424	43.2	
≥70	11,446	11.9	7,969	11.9	3,477	12.1	
**Race^a^**							0.247
White	78,440	81.9	54,968	82.0	23,472	81.7	
Black	12,182	12.7	8,524	12.7	3,658	12.7	
Other	5,190	5.4	3,580	5.3	1,610	5.6	
**Marital status**							0.110
Married	77,053	80.4	54,030	73.4	23,023	73.8	
Single^b^	18,759	19.6	13,042	21.1	5,717	21.2	
**Pathological extension**							0.080
Organ-confined	67,469	70.4	47,120	70.3	20,349	70.8	
Extracapsule-invasion	18,041	18.8	12,655	18.9	5,386	18.7	
Seminal vesicle invasion	9,371	9.8	6,658	9.9	2,713	9.4	
Adjacent structures invasion	931	1.0	639	1.0	292	1.0	
**Regional lymphonode status**							0.346
Negative	91,526	95.5	64,044	86.4	27,482	95.6	
Positive	4,286	4.5	3,028	13.6	1,258	4.4	
**PSA level (ng/ml)**							0.777
≤ 5.9	45,664	47.7	31,971	47.7	13,693	47.6	
6.0–14.9	39,882	41.6	27,886	41.6	11,996	41.7	
>14.9	10,266	10.7	7,215	10.8	3,051	10.6	
**GS**							0.131
≤ 6	23,522	24.6	16,444	24.5	7,078	24.6	
7 (3 + 4)	40,563	42.3	28,334	42.2	12,229	42.6	
7 (4 + 3)	16,887	17.6	11,809	17.6	5,078	17.7	
7(N/A)	108	0.1	73	0.1	35	0.1	
8	6,908	7.2	4,878	7.3	2,030	7.1	
9	7,581	7.9	5,379	8.0	2,202	7.7	
10	243	0.3	155	0.2	88	0.3	
**EAU risk classification**							0.455
Low risk	3,316	3.5	2,306	3.4	1,010	3.5	
Intermediate risk	5,250	5.5	3,654	5.4	1,596	5.6	
High risk	76,649	80.0	53,630	80.0	23,019	80.1	
Unknown	10,597	11.1	7,482	11.2	3,115	10.8	

a *Black: African American, White: Caucasian, Other: American Indian/AK Native, Asian/Pacific Islander*.

b *Single: Divorced, Separated, Single (never married), Widowed, unmarried*.

### Identification of Independent Prognostic Factors

We performed the analyses of CIF and Gray's test as the univariable analyses. The results showed that age, race, marital status, pathological extension, regional lymphonode status, PSA level, and pathological GSwere the factors with a significant impact on CSD. The Fine and Gray's proportional subdistribution hazard approach was performed as the multivariable analyses. And the results were consistent, in which age, race, marital status, pathological extension, regional lymphonode status, PSA level, and pathological GS were the significant prognostic factors of CSD. These variables could be thought of as the independent prognostic factor for predicting the CSS of PCa patients after RP. The detailed results of univariable and multivariable analyses were showed in Table 2.

**Table 2:** Univariate analyses and multivariate competing risk analyses of prognostic factors influencing cancer-specific survival outcomes in the training cohort.

**Variable**	**Univariate analyses (CIF)** ***p*****-value**	**Multivariate competing risk analyses sdHR (95%CI)**	***p*** **-value**
**Age**	<0.001		
≤ 60		Ref.	
61–69		1.098 (0.972–1.240)	0.132
≥70		1.308 (1.103–1.551)	0.002
**Race^a^**	0.024		
White		Ref.	
Black		1.225 (1.043–1.440)	0.014
Other		0.698 (0.529–0.920)	0.010
**Marital status**	<0.001		
Married			
Single^b^		1.253 (1.099–1.429)	<0.001
**Pathological extension**
Organ-confined		Ref.	
Extracapsule-invasion		2.273 (1.956–2.641)	<0.001
Seminal vesicle invasion		3.576 (3.039–4.207)	<0.001
Adjacent structures		4.245 (3.261–5.525)	<0.001
Invasion			<0.001
**Regional lymphonode status**	<0.001		
Negative		Ref.	
Positive		1.917 (1.647–2.231)	<0.001
**PSA level (ng/ml)**	<0.001		
≤ 5.9		Ref.	
6.0–14.9		1.252 (1.043–1.440)	<0.001
>14.9		1.430 (1.214–1.686)	<0.001
**GS**	<0.001		
≤ 6		Ref.	
7 (3 + 4)		1.647 (1.291–2.101)	<0.001
7 (4 + 3)		3.150 (2.441–4.065)	<0.001
7 (N/A)		3.980 (1.253–12.641)	0.019
8		5.065 (3.900–6.583)	<0.001
9		10.827 (8.448–13.874)	<0.001
10		20.528 (13.803–30.978)	<0.001

a *Black: African American, White: Caucasian, Other: American Indian/AK Native, Asian/Pacific Islander*.

b *Single: Divorced, Separated, Single (never married), Widowed, unmarried*.

*CIF, cumulative incidence function; sdHR, subdistribution hazard ratio; Ref., reference*.

### Development and Validation of the Competing Risk Nomogram

**Figure 3:**
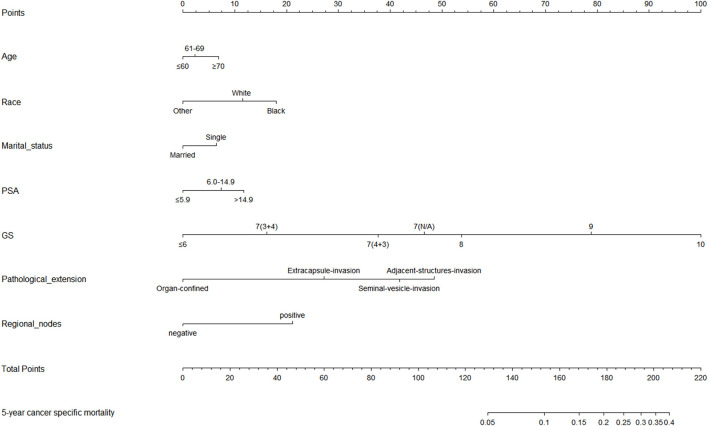
Nomogram for predicting the cancer specific mortality (CSM) at 5 years in prostate cancer patients undergoing radical prostatectomy. (1) Black: African American, White: Caucasian, Other: American Indian/AK Native, Asian/Pacific Islander. (2) Single: Divorced, Separated, Single (never married), Widowed, unmarried.

Based on the results of univariable and multivariable competing risk analyses, age, race, marital status, pathological extension, regional lymphonode status, PSA level, and pathological GS were used to construct the nomogram for predicting the probability of 5-year CSD for PCa patients after RP (Figure 3). The detailed score of each nomogram variable was listed in Table 3.

**Table 3:** Detailed risk scores of all independent prognostic factors in the nomogram.

**Variables** **Age**	**Nomogram risk score**	**Variables** **Regional lymphonode status**	**Nomogram risk score**
≤ 60	0	Negative	0
61–69	2	Positive	21
≥70	7	**PSA level (ng/ml)**	
**Race**		≤ 5.9	0
White	11	6.0–14.9	7
Black	18	>14.9	12
Other^a^	0	**GS**	
**Marital status**		≤ 6	0
Married	0	7 (3 + 4)	16
Single^b^	6	7 (4 + 3)	38
**Pathological extension**		7 (N/A)	47
Organ-confined	0	8	54
Extracapsule-invasion	27	9	79
Seminal vesicle invasion	42 at 5 years	10	100
Adjacent structures invasion	49		
**Total points**	**Predicted probability of 5-year CSM**		
154	0.10		
179	0.20		
195	0.30		
207	0.40		

*CSM, cancer specific mortality*.

**Figure 4:**
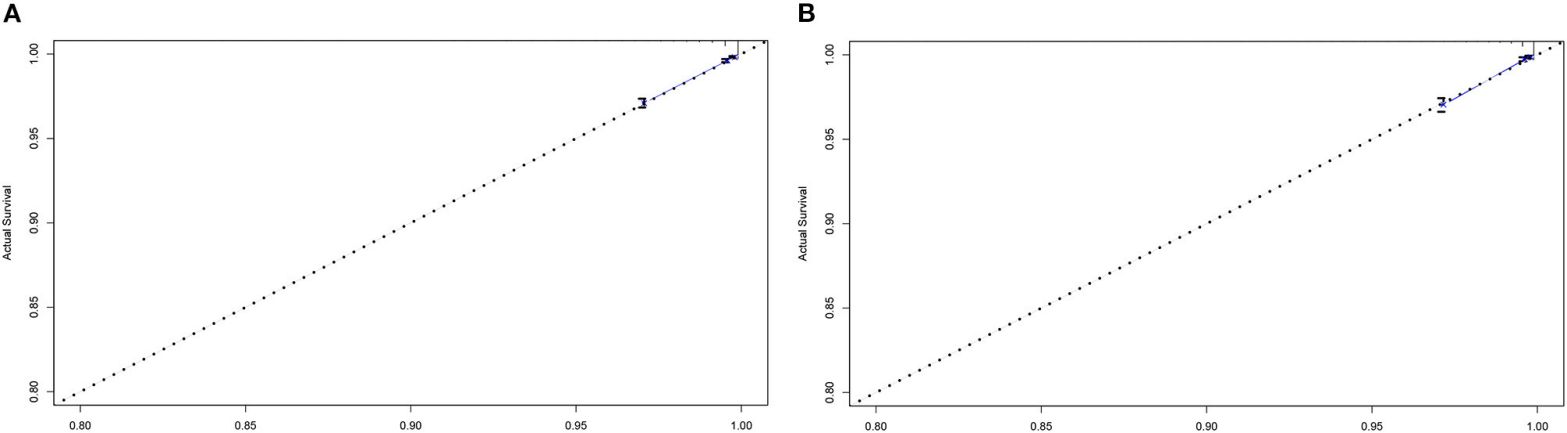
Calibration curves for comparing the degree of agreement between the actual survival outcome and the survival outcome predicted by the nomogram in the training cohort **(A)** and the validation cohort **(B)**. The horizontal axis is the survival rate predicted by the nomogram, and the vertical axis is the actual survival rate. The dashed line indicates the predicting survival rate completely fits the actual survival rate.

We performed the analyses of C-index and calibration curve to validate the reliability of the nomogram. For the training cohort, the C-index was 0.828 (%95CI, 0.812–0.844). For the validation cohort, the C-index was 0.838 (%95CI, 0.813–0.863). The relatively high C-index (>0.8) showed the good predictive ability of this nomogram. Meanwhile, in both training cohort and validation cohort, the calibration curves showed a good agreement between the 5-year CSD predicted by nomogram and actual 5-year CSD (Figure 4).

### Establishment of Risk Stratification for Cancer-Specific Death After RP

According to the score corresponding to each nomogram variable, we calculated the total risk score for each patient in both the training cohort and the validation cohort. By the X-tile approach, patients were divided into three risk groups based on the total risk score from the nomogram. The low-risk group included patients with 0–66 points, the middle-risk group included patients with 67–105 points, and the high-risk group included patients with no <106 points.

**Figure 5:**
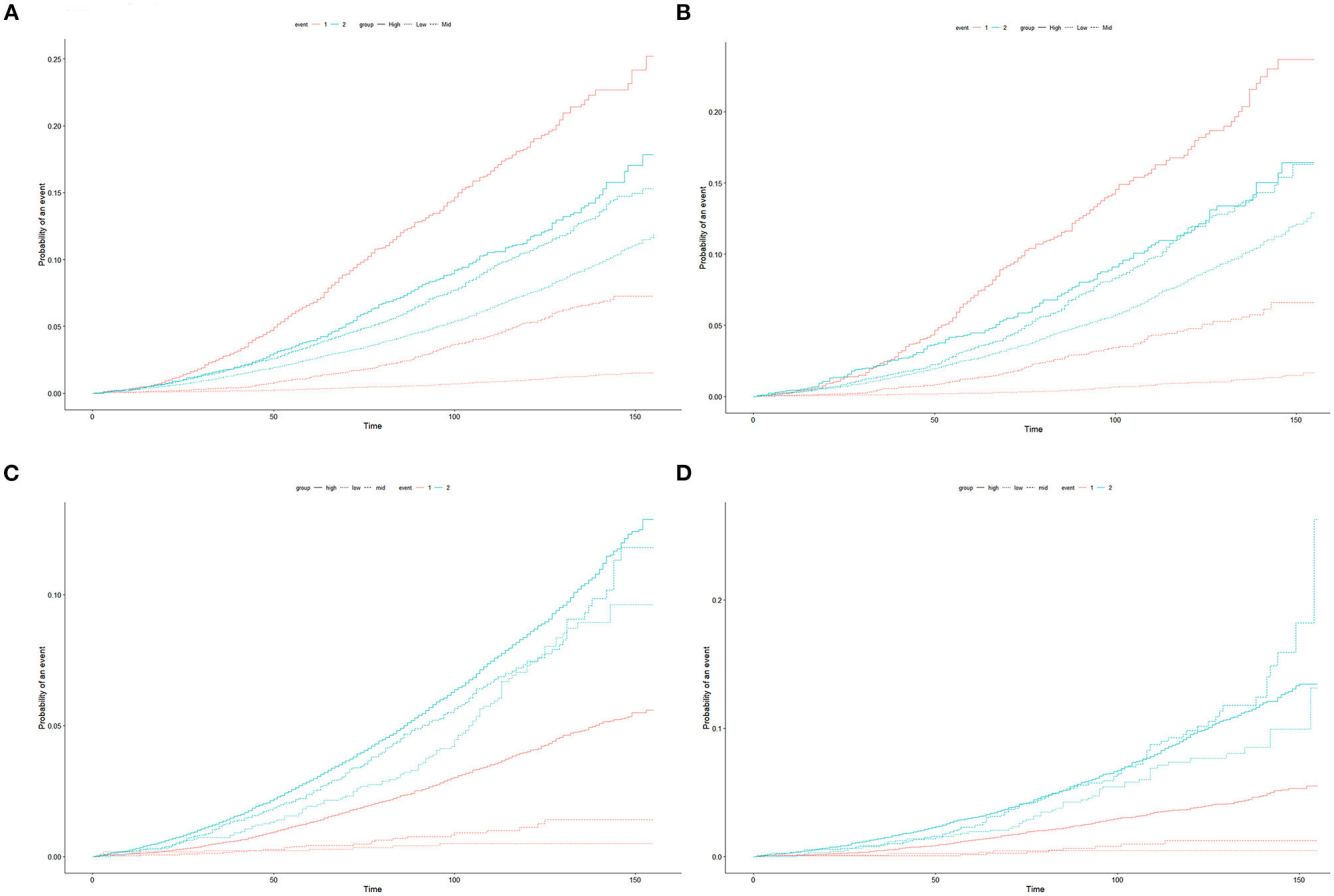
Cumulative incidence function (CIF) curves of different risk stratification systems for cancer specific mortality (CSM) in prostate cancer patients undergoing surgery. Nomogram risk stratification and EAU risk stratification based on D'Amico risk stratification were tested in the training cohort **(A,C)** and validation cohort **(B,D)**. As event 1, cancer-specific death was represented by red lines, and other cause-specific death was represented by event 2, and blue lines.

In order to verify the predictive value of this risk stratification, we compared it with the EAU risk stratification based on D'Amico stratification. EAU risk stratification is one of the most popular risk stratification tools for PCa patients, which can divide patients into three groups including low-risk, medium-risk, and high-risk. In both training cohort and validation cohort, we plotted the CIF curves for different risk groups based on the risk stratification of the nomogram or EAU risk stratification (Figure 5). Compared with our risk groups, the degree of separation of CIF curves of CSD between groups was more obvious than EAU risk stratification. Meanwhile, in both two cohorts, The high-risk group identified by our risk stratification had a significantly higher CSD risk than OCSD, while the high-risk group in the EAU risk stratification did not. The results showed that the novel risk stratification based on the nomogram had better prognostic discrimination than EAU risk stratification.

## Discussion

In this study, based on a large cohort of 95,812 patients from SEER database, we identified seven risk factors and construct a competing risk nomogram based on these prognostic factors to predict the probability of the occurrence of CSD within 5 years after RP for each patient with PCa. Furthermore, based on the difference in nomogram scores, we developed a novel risk stratification for post-operative CSD in patients with PCa. Our risk stratification has potential clinical value and may help clinicians better identify patients who still need active intervention after RP. The results showed that the discrimination of our stratification system was not weaker than the commonly used EAU risk stratification based on D'Amico stratification.

Competing risk nomogram is a kind of widely used risk predicting model in many fields in oncology such as lung cancer, breast cancer, and colorectal cancer (18–20). The nomogram can incorporate many key factors of the disease into the prognosis prediction model and can consider the weight of each variable to make the prediction model more accurate. In addition, the graphical representation helps to more intuitively evaluate the individual situation of each patient, which is more practical (21). At the same time, competing risk nomogram has its unique advantages compared to traditional nomogram or other prognosis predicting models. The competitive risk nomogram is based on competing risk analysis methods such as CIF and Fine and Gray's proportional subdistribution hazard approach, rather than the Kaplan-meier method and Cox proportional risk regression commonly used in other types of models. Competitive risk analyses not only consider the survival and death of patients but also consider the impact of death caused by other factors on the endpoints of interest such as CSD. This is especially important in the research of PCa, because a large part of PCa patients may die due to other factors before developing CSD (13). To our knowledge, there is still no research reported on the competitive risk prognosis prediction model for the prognosis of PCa patients after RP.

In the field of PCa, the currently commonly used nomogram is Stephenson nomogram. It is developed by Stephenson et al. to predict disease progression after salvage radiotherapy (SRT), with data from a multi-institutional retrospective cohort of 1,540 patients. Seven variables were used to construct the nomogram including PSA before SRT, surgical margins, GS, PSA double time before SRT, lymph node metastasis, and androgen deprivation therapy administration before or during SRT (22). However, there are still some defects with Stephenson nomogram. Due to the limitation of inclusion and exclusion criteria, it is not widely applicable to PCa patients who have received RP. At the same time, it paid little attention to hard endpoints such as CSD. In the cohort of the original study, its c-index was 0.69 (compared to 0.83 of our nomogram), and the c-index obtained after the test in another study was even lower (23). Another post-operative nomogram proposed by Cleveland Clinic in recent years has become more and more widely used in clinical practice (24). This nomogram mainly consisted of PSA level at the time of biochemical recurrence (BCR), pathological GS, seminal vesicle invasion, extraprostatic extension, preoperative PSA, and time to BCR. This study is based on the population of PCa patients with BCR after surgery and can predict the probability of patients eventually developing CSD. In the internal validation of 2,254 patients in the study, the c-index of the nomogram was 0.74, which was lower than the C-index of 0.83 obtained by our proposed nomogram in a cohort of 67,072 patients. At the same time, the clinicopathological parameters required by our nomogram can be obtained within a short period after surgery. Therefore, our nomogram has advantages in guiding patients' initial post-operative management compared with this nomogram that incorporates PSA dynamic parameters.

In our study, the competing risk analyses identified 7 prognostic factors including age, race, marital status, pathological extension, regional lymphonode status, PSA level, and pathological GS. Among them, GS had the greatest influence on survival outcomes. Many studies have reported the relationship between GS and the prognosis of PCa (25–27). International Society of Urological Pathology (ISUP) reported that GS can be divided into five groups [2–6, 7 (3 + 4), 7 (4 + 3), 8, ≥9] according to prognosis, and this was consistent with our research results (27). With the increase of GS, the patient's nomogram score was also increasing, that is to say, the possibility of the patient developing CSD within 5 years was increasing. In the nomogram, we could find that GS 4 + 3 = 7 group was with an obviously higher score than GS 3 + 4 = 7 group. This was also consistent with the latest American Urological Association (AUA) clinical guideline, which indicated that many pieces of research had demonstrated that the prognosis of GS 4+3 was significantly worse than GS 3 + 4 (28, 29). The pathological extension was another important prognostic factor whose weight was second only to GS. It has been widely accepted that poor pathological findings such as extracapsular invasion and seminal vesicle invasion are related to disease recurrence and poor prognosis (30–32).

In addition to the above-mentioned well-known prognostic factors, our study also found the impact of race and marital status on the prognosis of PCa patients. Our nomogram showed that African Americans had the highest risk of CSD after RP, followed by Caucasian and other races. This finding was consistent with some studies published in recent years. According to statistics from researchers, the average annual incidence of PCa among African Americans was 60% higher than that of Caucasian men. Besides, compared with other races, African Americans have the highest mortality rate (33, 34). The causes of the result were very complicated. For example, In the United States, PCa tended to be larger in African Americans and was more likely to metastasize than white men (35). From a genetic perspective, some gene mutations related to disease progression are more common in African Americans, such as TP53 mutations and MYC amplification (36). Several risk-associated single nucleotide polymorphisms were found to be overexpressed in African Americans (37). At the same time, African Americans may face some social barriers such as health insurance, which may affect the treatment and management of the disease (38). Our competing risk analyses also identified marital status as an independent prognostic factor. More and more researchers have paid attention to the impact of this sociological factor on the disease. Outcomes of numerous studies showed that married marital status was a protective factor for the occurrence and development of a variety of tumors, including PCa. Marriage may be a multifaceted representation of many protective factors including social support (39, 40).

EAU risk stratification based on D'Amico stratification is currently a common risk stratification system for PCa patients, which divided patients into Low-risk group, Intermediate-risk group, and High-risk group for predicting the risk of disease recurrence (2, 41). In our study, we compared the novel risk stratification based on the nomogram with EAU risk stratification. The results showed that our risk stratification system had better discrimination with a C-index over 0.8 and could better detect patients at higher risk of the occurrence of post-operative CSD after adjustment of competing risk analyses. The high-risk group obtained through our risk stratification had a significantly higher risk of CSD than OCSD, which could better exclude the interference of death caused by non-tumor factors on the model. Our advantages may come from many aspects, such as a large cohort, more prognostic factors, and independent analyses of competing risks. At the same time, our research provides quantitative and graphical prognostic tools, which help to make more accurate assessments of each patient.

Our study revealed 7 main independent prognostic factors that affect the occurrence of CSD in patients after RP and explored the application of these factors in identifying high-risk patients through the nomogram and risk stratification. At present, the guidelines pointed out that there were multiple managements for patients undergoing RP, including adjuvant treatment, salvage treatment, watchful waiting, etc. However, due to the lack of high-quality prospective data, the inclusion and exclusion criteria of patients are still controversial (2). Although radical surgery has been implemented, many men may still need adjuvant or salvage treatment to prevent or delay clinical metastasis, thereby reducing the likelihood of patients dying from tumors (42). However, because the frequency of clinic5al metastasis or death in PCa patients is generally low, the choice of adjuvant therapy in all men may mean a large amount of overtreatment. Moreover, adjuvant therapy has obvious side effects. Adjuvant radiotherapy is an independent predictor of urinary incontinence and intestinal dysfunction, and androgen deprivation therapy may further aggravate post-operative erectile dysfunction (42, 43). Therefore, good prognostic stratification tools after RP to wisely guide the use of adjuvant therapy is urgently needed. In 2019, the EAU prostate cancer guidelines update proposed that the risk of subsequent metastasis after prostate cancer surgery, as well as PCa CSD and overall mortality, can be determined by initial clinical and pathological factors (Tstage, ISUP grade) and PSA dynamics (PSA double time and PSA failure interval) to predict (44). This update is consistent with our findings that ISUP grade is significantly associated with important clinical endpoints after prostate cancer surgery. However, because data of PSA kinetics was obtained from long-term follow-up after surgery, the application of PSA kinetics in formulating short-term patient management strategies after surgery is limited. Our research mainly includes data that can be easily obtained in a short period after surgery to help clinicians consider the patient's post-operative management plan promptly. For example, in our risk stratification system, patients in different risk groups have significantly different clinical endpoints. For high-risk patients, more aggressive post-operative management strategies, such as the use of adjuvant therapy after surgery, may be required. For patients in the low-risk group, the present study provides insights into which men can adopt more conservative post-operative management strategies, such as watchful waiting.

The prognostic model proposed in our study incorporates the most basic and most accessible clinicopathological information, making it applicable to almost all urological tumor centers. In addition, the application of some new technologies can bring more accurate clinical information to improve our clinical management. In recent years, the role of multiparametric magnetic resonance imaging (mpMRI) in the diagnosis and prediction of prognosis for PCa patients has received extensive attention from researchers. It is reported that mpMRI can significantly reduce the number of unnecessary repeat prostate biopsies (45). Some researchers have tried to use mpMRI to determine risk stratification for PCa patients (46). The results showed that the fusion of mpMRI data and clinicopathological data can significantly improve the predictive model's ability to predict the recurrence of PCa disease, compared to traditional predictive models that only have clinical data. Prostate-specific membrane antigen positron emission tomography (PSMA-PET) is another novel PCa visualization technology, which is characterized by high-precision visualization of primary PCa masses and provides superior accuracy at initial staging. At the same time, in a small-scale cohort, researchers reported the high accuracy of PSMA-PET-derived radiomics features for the diagnosis of visually unknown PCa (47). A newly reported study proposed a nomogram combining clinical information and PSMA-PET information. In the high-risk prostate cancer population cohort, the results showed that the fusion prognostic prediction model had an important prognostic effect on important clinical endpoints, and its performance was better than currently used prognostic tools (48). Some studies on the prognosis of PCa have made progress at the genetic level. For example, PCa susceptibility variants are found to be closely related to the prognosis of PCa, called single nucleotide polymorphisms, which are mainly involved in tumor cell invasion (49). The presence or absence of DNA repair-related changes in BRCA1 or BRCA2 has been reported to have a significant impact on the clinical endpoint after radical treatment of PCa (50). Evaluation of the TMPRSS2:ERG fusion gene, PTEN, the Prolaris test, the Decipher test, and the Oncotype DX Genomic Prostate Score can also provide important information helping improve the individual management for PCa patients (51). Some studies have established prognostic models based on the expression of immune-related genes and autophagy-related genes, which had considerable accuracy in predicting BCR (52, 53). However, at present, due to the lack of equipment, funds, and technical personnel, the implementation of mpMRI, PSMA-PET, and genetic evaluation are still limited (54). Our prediction model still has relatively high accuracy even when only common clinicopathological parameters are included, so our research results may be more practical. In the future, an improved prognostic prediction model that integrated these novel technologies will be able to better guide accurate clinical decision-making, and it will also be the goal of our team's future exploration.

There are still several limitations to our study. First, our research is based on a large retrospective cohort. We still need more prospective clinical trials to contribute more precise data. Second, in the data included in our study, there is a lack of data on the use of adjuvant therapy. This is due to the data limitation of the database. Our study included nearly 100,000 patients, and a large number of study populations made it difficult to follow up patients with post-operative adjuvant therapy. Many studies on the risk factors of prostate cancer recurrence after surgery have not included information about the adjuvant treatment of patients (24, 55). In current randomized trials and observational studies, there are conflicting data on the effects of early post-operative radiotherapy and early androgen deprivation therapy on CSD (24). Our risk stratification system performed well in validation, and the lack of adjuvant treatment data had little impact on our research results. Although based on the SEER database, many research teams have developed a series of well-known clinical prognosis prediction models, but the SEER database still has some inherent limitations (56–58). The lack of adjuvant treatment records, changes in data reports, patient migration, and selection bias are some of the problems in large-scale real-world studies based on the SEER database. In the future, we look forward to construct a relatively small prospective cohort and try to use these parameters to further optimize the nomogram and risk stratification system. Finally, we also lack additional independent external validation sets, and this is our important work goal in the future.

## Conclusions

In conclusion, we performed a competing risk analysis based on a larger cohort of 95,812 patients with non-metastatic PCa from the SEER database. We also identified 7 independent prognostic factors of the occurrence of CSD after RP and constructed a competing risk nomogram utilizing the 7 factors for detecting the risk of CSD for each patient. A risk stratification system was established based on the nomogram to help clinicians better identify patients at high risk of CSD after surgery.

## Data Availability Statement

The original contributions presented in the study are included in the article/supplementary material, further inquiries can be directed to the corresponding author.

## Ethics Statement

Ethical review and approval was not required for the study on human participants in accordance with the local legislation and institutional requirements. Written informed consent for participation was not required for this study in accordance with the national legislation and the institutional requirements.

## Author Contributions

XZho, KJ, SQ, QY, QW, and LY are responsible for designing the study, writing, collecting data, analysis, and final approval of the article. DJ and KJ are responsible for a part of analysis and revision. ZZ, XZhe, and JL are responsible for a part of writing and revision. All authors have read and approved the final manuscript.

## Funding

This work was supported by the National Key Research and Development Program of China (Grant No. 2017YFC0908003), National Natural Science Foundation of China (Grant Nos. 81902578 and 81974098), China Post-doctoral Science Foundation (2017M612971), Post-doctoral Science Research Foundation of Sichuan University (2020SCU12041), Post-Doctor Research Project, West China Hospital, Sichuan University (2018HXBH085), National Clinical Research Center for Geriatrics, West China Hospital, Sichuan University (Z2018C01).

## Conflict of Interest

The authors declare that the research was conducted in the absence of any commercial or financial relationships that could be construed as a potential conflict of interest.

## Publisher's Note

All claims expressed in this article are solely those of the authors and do not necessarily represent those of their affiliated organizations, or those of the publisher, the editors and the reviewers. Any product that may be evaluated in this article, or claim that may be made by its manufacturer, is not guaranteed or endorsed by the publisher.

## Abbreviations

PCa: Prostate cancer
CI: confidence interval
RP: radical prostatectomy
SRT: salvage radiotherapy
GS: Gleason Score
CSS: cancer-specific survival
CSM: cancer-specific mortality
CIF: cumulative incidence function
PSA: prostate specific antigen
SEER, the Surveillance, Epidemiology: and End Results
CSD: Cancer-specific death
OCSD: Other cause-specific death
EAU: European Association of Urology
ISUP: International Society of Urological Pathology
AUA: American Urological Association.
